# Ministry of Health Estimates

**Published:** 1923-05

**Authors:** 


### THE MINISTRY OF HEALTH ESTIMATES.

THE Estimates of the Ministry of Health for 1923-4, including those of the Welsh Board of Health, amount to £19,504,210, and show a decrease as compared with 1922-3 of about £3,000,000. The Estimates of the Scottish Board of Health amount to £2,493,730, and shew an increase, as compared with the previous year, of £781,308. Our readers will not fall into the error, sometimes observed in the daily press, of supposing that these amounts represent the cost of the central administration, and will appreciate that they include the Exchequer Grants for Housing, National Health Insurance and various special Health Services. One source of difficulty in appreciating the real significance of figures contained in the estimates of public expenditure is that these estimates are on a cash basis, or, in other words, deal with the payments falling to be made within the financial year, and not to expenditure accruing due within the year. This fact will explain, for example, such an apparent anomaly as the increase in the Scottish Estimates compared with the large decrease in those for England and Wales.

### Housing Grants.

The difference just referred to can be traced in large part to the fact that the grants towards deficits on housing schemes are down in England by about a million pounds, while in Scotland they shew a substantial increase of over half a million. These grants are in connection with the schemes where the Exchequer undertook to bear annual expenditure in excess of a penny rate. The increased provision in the Scottish estimates would appear to imply not any preferential treatment for Scotland, but merely some delay in the housing schemes or in the presentation of the accounts of the local authorities. The subsidy to private builders for which £2,500,000 was provided in the 1922-3 Estimates for the Ministry of Health now disappears, and the corresponding Scottish estimate shows a decrease of about £400,000. The figures for housing are affected also by the virtual disappearance of the receipts for sales of building materials shown in the estimates as appropriations in aid. The net housing figures considered as a whole account for a decrease of about 2½ millions, out of the total decrease of 3 millions in the English and Welsh Estimates, and for an increase in the Scottish Estimates equivalent to the total amount of the increase therein.

### Unemployment.

It is something of a surprise to come across an entry in the Ministry of Health Estimates for grants in respect of Unemployment schemes. The provision for 1923-4 is £1,100,000, shewing an increase of £650,000 over the figures for the previous year. This provision is for the annuity payment to local authorities in connection with their approved loan schemes, and must be differentiated from the payments for Unemployment Insurance, £13,000,000, in the Estimates of the Ministry of Labour; although in considering the total Exchequer assistance for Unemployment it must be read in conjunction with that amount, and also with the provision of £2,500,000 in the Unclassified Services in the Estimates.

### National Health Insurance.

The Estimates for Sickness, Disablement and Maternity Benefits in Great Britain show a decrease from £4,900,000 to £4,300,000. These amounts, of course, do not represent the total cost of these benefits, but only the Exchequer Contribution to Insurance funds. The decrease of about one-sixth is not to be explained by an estimated drop in the sickness experience of 1923 as compared with 1922, but by the fact that an over-estimate appears to have been made in the year 1922 of the extent to which sickness would make demands upon the funds in that year.

### Miscellaneous Health Grants.

These comprise in the main Maternity and Child Welfare, Treatment of Tuberculosis, Grants in respect of Sanatoria, Treatment of Venereal Diseases, and Grants for the Welfare of the Blind. The estimated grants for these services amount in the aggregate for Great Britain to about 3½ millions for 1923-4, as compared with 3¾ millions for 1922-3. The general policy of the Department in regard to these services during the present period of financial stringency would appear to be that of curtailing developments, and securing the greatest possible efficiency for existing expenditure.

---

### HOSPITALS EXTENDING THEMSELVES.

### University College Hospital.

The King and Queen will lay the foundation stones of the new buildings presented by the Rockefeller Foundation to University College Hospital at the end of May. The new obstetric hospital will be inaugurated by the King and the new nurses' home by the Queen. The maintenance of the new buildings will cost £20,000 a year, and Sir Ernest Hatch has explained that the charitable side of the work will not benefit by the gift from America.

### St. Luke's Hospital for Advanced Cases.

The lease of the house in Pembridge Square occupied by St. Luke's Hospital for Advanced Cases, Bayswater, will expire in December. Even if the house had not been outgrown, the tenancy could not be renewed. A freehold site has been obtained near by, between Pembridge Square and Hereford Road, in the garden of which a hospital of 48 beds could be built. The adjoining house would form the nurses' home. A sum of £10,000 is still needed to re-establish the hospital in its new site. The value of St. Luke's to other hospitals, and so to the sick public, is shown in this, that more than half its patients come from other hospitals which cannot retain them.

### Colwyn Bay Hospital.

The Colwyn Bay and District Cottage Hospital will shortly be known as the Colwyn Bay and West Denbighshire Hospital, when the new building, for which the contract has been signed, is finished. The new hospital will probably be complete before the end of the year.

### Victoria Hospital, Belfast.

Three new wards, containing 60 beds, an extension of the out-patient department, and a new nursing block are to be added to the Royal Victoria Hospital, Belfast, at an estimated cost of £64,000.

